# Diverse Morphology and Structural Features of Old and New World Hantaviruses

**DOI:** 10.3390/v11090862

**Published:** 2019-09-16

**Authors:** Amar Parvate, Evan P. Williams, Mariah K. Taylor, Yong-Kyu Chu, Jason Lanman, Erica Ollmann Saphire, Colleen B. Jonsson

**Affiliations:** 1 Department of Biological Sciences, Purdue University, West Lafayette, IN 47906, USA; aparvate@lji.org (A.P.);; 2 Department of Microbiology, Immunology and Biochemistry, University of Tennessee Health Science Center, Memphis, TN 38163, USA, mtayl121@uthsc.edu (M.K.T.); 3 Center for Predictive Medicine for Biodefense and Emerging Infectious Diseases, University of Louisville, Louisville, KY 40202, USA; yongkyu.chu@louisville.edu; 4 La Jolla Institute for Immunology, La Jolla, CA 92307, USA; erica@lji.org

**Keywords:** *Orthohantavirus*, *Hantaviridae*, cryo-electron microscopy (EM), Old World hantaviruses, New World hantaviruses, Andes (ANDV), Sin Nombre (SNV), Black Creek Canal (BCCV)

## Abstract

To further understanding of the structure and morphology of the *Orthohantavirus*, family *Hantaviridae*, we have employed cryo-electron microscopy (cryo-EM) for three New World hantaviruses: Andes (ANDV), Sin Nombre (SNV), and Black Creek Canal (BCCV). Building upon our prior cryo-EM and cryo-tomography study of the Old World hantavirus, Hantaan virus (HTNV), we have expanded our studies to examine the entire virion population present in cell culture supernatant. Hence, in contrast to the prior cryo-EM/ET studies in which we used a polyethylene precipitation, a sucrose gradient, and a sucrose cushion, we used two sucrose cushions. We inactivated the material after the first cushion. We tested the method using HTNV which has a known cryo-EM structure and observed equivalent results. Therefore, we used this method to assess the particle distribution of the New World hantaviruses by cryo-EM. Cryo-EM images showed a diverse range of sizes and morphologies for the New World viruses that we classified as round, tubular, and irregular. Strikingly, BCCV virions were mostly tubular. These first cryo-EM images of the New World *Orthohantavirus* confirm prior EM observations that noted tubular projections of SNV at the plasma membrane during virion morphogenesis but were not confirmed. These findings underscore the need for further investigation of virion morphogenesis of the *Orthohantavirus*.

## 1. Introduction

Hantaviruses, genus *Orthohantavirus*, Family *Hantaviridae*, Order *Bunyavirales*, are widely distributed in nature in rodents, bats, shrews, and moles [1]. Species of hantaviruses have been discovered on all continents except Australia and Antarctica, and some species of rodent borne virus have been linked to severe human disease in Europe, Asia, and the Americas. Hantaviruses endemic to Eurasia are referred to as Old World hantaviruses and include pathogens that cause hemorrhagic fever with renal syndrome (HFRS), whereas hantaviruses endemic to the Americas are referred to as New World hantaviruses and include pathogens responsible for hantavirus pulmonary syndrome (HPS). The mortality of HFRS and HPS cases range from 10% to 40% with Old World viruses having less lethality (<1 to 10%) [2]. There are no vaccines or antivirals approved for treatment or prevention of HRFS or HPS [3]. 

Hantaviruses are negative-sense, single-stranded RNA viruses with three gene segments, small (S), medium (M), and large (L), which encode the nucleocapsid, Gn and Gc glycoproteins and the RNA-dependent RNA polymerase, respectively [4]. Cryo-electron microscopy (cryo-EM) studies of two Old World viruses, Hantaan virus (HTNV) and Tula virus (TULV), reported that virion particles have predominantly round, pleomorphic morphology [5,6]. Both studies noted particles of tubular morphology but do not describe them further [5,6]. Prior cryo-EM and cryo-tomography (cryo-ET) structural studies of these viruses show the particles range in size from 120–154 nm with an average diameter of 135 nm [5,6]. Moreover, these studies show the virion has a surface structure composed of a square, grid-like pattern distinct from other families within the *Bunyavirales*, such as Rift valley fever virus [7] or Uukuniemi virus [8], in which the glycoproteins form pentavalent and hexavalent structures. The square surface projections of the Old World viruses are formed from a tetramer of Gn/Gc glycoprotein spike proteins that extends approximately 10 nm from the lipid bilayer [5], derived from the Golgi. Internally, the virion contains three rod-like ribonucleoprotein (RNP) structures which presumably contain one segment each of the viral RNA genome wrapped in N proteins [9].

Currently, there are no cryo-EM studies reporting the ultrastructure of New World hantaviruses. This may be due to the scarcity of cryo-EM microscopes in biosafety level-3 (BSL-3) facilities, or conversely, the lack of established methods to inactivate virions for removal from BSL-3 for imaging at BSL-1/2. To enable such studies, we have developed and verified a method for inactivation and preservation of virion structure. The method eliminates two steps we have used to purify hantaviral particles for cryo-EM in our prior publication [5], overnight polyethylene glycol precipitation (PEG) at 4 °C and ultracentrifugation, thus greatly reducing the effects of purification on virion morphology. Instead, we used two gentle low-speed centrifugation steps which enabled remarkable concentration and purification of the virions [10]. Here, we employed the same strategy to concentrate BSL-3 hantaviruses for cryo-EM analysis of the Old World, HTNV, and New World hantaviruses, Sin Nombre virus (SNV), Black Creek Canal virus (BCCV), Andes virus (ANDV). We report that the resulting cryo-EM images of glutaraldehyde-fixed HTN virions resemble those observed in previous studies of HTNV and TULV [5,6,11] and provide a robust approach for the cryo-EM of the New World hantaviruses. Herein, we report a comprehensive assessment of the morphology and particle type distribution for HTNV, SNV, ANDV, and BCCV. All viruses adopted three distinct morphology groups—round, tubular, and irregular morphology. The Old World HTNV, however, had a greater percentage of round particles relative to previous studies, and New World SNV, BCCV, and ANDV exist in a greater propensity of tubular and irregular virion morphologies. The diversity of structures is intriguing and provides new and additional insight into the diverse morphology of the genus, *Orthohantavirus*. 

## 2. Materials and Methods

### 2.1. Cells, Virus Propagation, and Virus Inactivation

Virus strains used in these studies were HTNV 76-118, ANDV isolate Chile-9717869, BCCV isolate 807040, SNV strain Muerto Canyon (MCV), and SNV strain Convict Creek 107 (CC107). Hantaviruses were amplified in Vero E6 cells in Minimal Essential medium (EMEM) supplemented with 10% fetal bovine serum (FBS) and 5 mM penicillin/streptomycin, incubated at 37 °C, 5% CO_2_ for 7 or 10 days, concentrated and inactivated at BSL-3 at the Regional Biocontainment Laboratory at the University of Louisville or University of Tennessee Health Science Center prior to removal. Briefly, four T175 flasks containing Vero E6 cells were infected at a MOI of 0.1. Viruses were inactivated and purified as described in [10]. Briefly, 200 mL of supernatant was pooled from four T175 flasks and centrifuged for 15 min at 3000× *g*. The supernatant was transferred to a new 50 mL conical and centrifuged again for 15 min at 3000× *g*. The clarified supernatant was layered on top of a 30% sucrose cushion and centrifuged for 8 h at 10,000× *g* in a fixed angle rotor at 4 °C. Pelleted material was resuspended in a total volume of 1.54 mL of HNE pH 7.2 (0.1 M HEPES, 100 mM NaCl and 0.5 mM EDTA) and allowed to resuspend on ice for 15 min to overnight. Virus-containing supernatant was inactivated by bringing the suspension to a final *v*/*v* of 1% glutaraldehyde and placed on ice, in closed tubes wrapped in aluminum foil. The material was shipped overnight on ice blocks to Purdue University. On arrival, the samples were pelleted through a second 30% sucrose cushion. The pellet was resuspended in 100 µL TNE buffer and processed for cryo-EM. 

To verify inactivation, supernatant from HTNV-infected Vero E6 cells that were grown on 6 well plates for 7 days was harvested. Samples were treated with a final volume of 1% glutaraldehyde, or vehicle, and incubated for 30 min. Inactivated or active virus culture was diluted serially, 10-fold in the DMEM cell culture media followed by inoculation and cultivation in Vero E6 cells in a 96-well plate. A week later, inoculated cells were fixed with acetone-methanol (1:1) mixture and air-dried. Cells were probed for virus by ELISA using polyclonal immune serum to the glycoprotein of HTNV. As compared to the virus-infected Vero E6 cells, no virus was detected. In the untreated samples, titers of greater than 10^5^ were observed.

### 2.2. Cryo-Electron Microscopy

For cryo-EM analysis, 3.5 µL of the virus samples were loaded onto glow discharged Holey Carbon grids (CF 1.2/1.3-2C, EMS). The grids were blotted for 6 s prior to plunging into liquid ethane using Gatan Cryoplunge™ 3 (Gatan, Inc., Pleasanton, CA, USA). The grids were stored under liquid nitrogen until further use. Blotting times and ice thickness for all viruses were optimized to suit the different sizes of all observed particles. Grids were loaded on to a CM200 (FEG Phillips) electron microscope or a FEI Titan Krios to screen for the presence of virus particles. Ice thickness was estimated for every grid as per the method by Yan et al. [12]. Projection images were obtained for different viruses at 38,000×, 50,000× or 14,000× magnifications at defocus of −3 to −5 μm using a 4K × 4K CCD camera or K2 Summit camera. ImageJ was used to process the cryo-EM images to better visualize the virus particles and to measure the dimensions of the particles. Using ImageJ, dimensions were estimated for virions of each species to obtain an estimate of the morphological distribution (number of virions included in the analyses were 37 for HTNV, 216 for ANDV, 56 for BCCV, 31 for CC107, and 34 for MCV.).

### 2.3. Cryo-Electron Tomography

Virus samples were mixed with 10 nm gold bead solution at a 5:1 ratio. About 3.5 µL of the virus sample was loaded on glow discharged, 200 mesh Holey Carbon grid, blotted for 6 s and plunge frozen. For tomography, tilt series were collected on an FEI Titan Krios using Leginon at a nominal magnification of 11,000×, on a K2 Summit camera, from −60 °C to +60 °C in 2° increments, with a total dose varying between 60–100 e^−^/Å^2^/s. Tomograms were collected in super resolution mode at a pixel size of 0.65 Å, at a defocus of −3 to −4 um, using a dose symmetric collection scheme. A python script for motion correction was run in parallel, which also sorted the images from negative to positive tilts. Tilt series were binned 4× before processing. Reconstruction was carried out in IMOD using both SIRT and weighted back projection method. The SIRT reconstruction was further processed by Non-Linear Anisotropic Diffusion filtering to enhance contrast.

## 3. Results

### 3.1. Morphology of Hantaan Orthohantavirus 

Our prior studies reported the first cryo-EM and cryo-ET of HTNV [5], in which we purified HTNV particles from a single band prepared using a 20% to 60% sucrose gradient and high-speed ultracentrifugation. In the current study, we used a simple and gentle purification strategy [10], wherein following two low speed centrifugation of material on a 30% sucrose cushion, the pelleted virions were resuspended, fixed in 1% glutaraldehyde and inactivated. The second sucrose cushion removed excess glutaraldehyde which might be a source of noise in cryo-EM mages. This eliminated two steps used in our prior purification of HTNV, an overnight PEG precipitation step and ultracentrifugation of this material on a sucrose gradient. Hence, this eliminated substantial manipulation of the material. Herein our goal was to examine virion structure of New World hantaviruses as compared to HTNV. To enable this comparison and to explore the intrinsic pleotropic nature of these viruses, we used HTNV as a benchmark and hence describe results for this virus first. In development of the method, we estimated a titer 10^5^ to 10^6^ PFU of per ml for HTNV-infected Vero E6 cells supernatant. Hence, we estimated a final yield of 2 × 10^7^ to 10^8^ virions from 200 mL media harvested. We typically observed an average of 1–2 virions observed per hole on the cryo-EM grids. 

**Figure 1:**
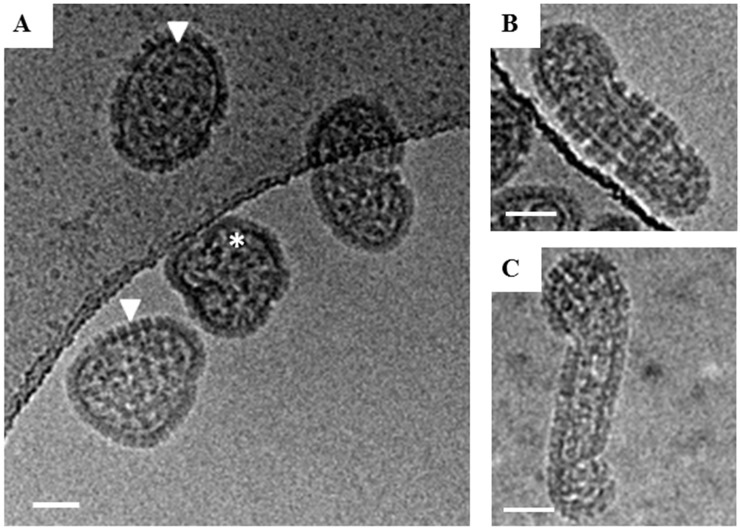
Cryo-EM images of glutaraldehyde-fixed HTNV particles. Examples of the three classes of virions observed in Holey Carbon grids by cryo-EM are presented. (**A**) Examples of round HTNV particles are indicated by a white triangle. The Asterix denotes an irregular shaped, crumpled ball. (**B**) An example of a tubular particle, which were those with their length at least twice their diameter. (**C**) An example of an irregular particle with a hybrid of round and tubular portions. Scale bar = 50 nm.

**Figure 2:**
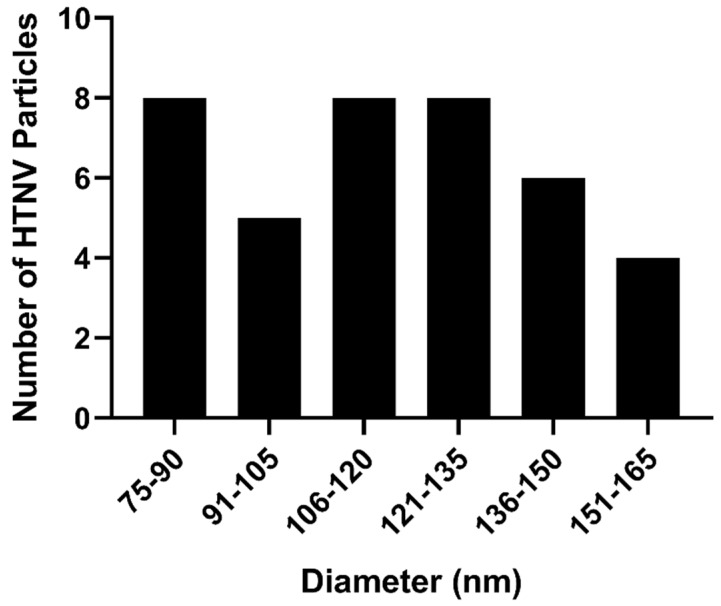
Histogram of diameters measured for HTNV virions with round morphology. The diameters of 37 virions with a round morphology were measured from several cryo-EM images.

We analyzed the morphologies of at least 100 HTNV particles by cryo-EM. We estimated and optimized ice thickness for each virus keeping in mind the diverse range of sizes Yan et al. [12]. About 65% of the particles were round with a mean diameter of 118 nm over a range of 88–148 nm (Figure 1A). Our average diameter is smaller than the previously reported for sucrose-gradient purified virus for which the average diameter for most round particles was 130–134 nm [5]. For those particles that had their length at least twice their diameter, we classified them as tubular (Figure 1B). The mean length by width of this class of particles was 176 ± 31 nm by 76 ± 10 nm Particles which could not be distinguished as either round or tubular were classified as irregular (Figure 1C). In summary, we observed three basic morphologies, round (65%) (Figure 2), tubular (30%) and irregular (5%) (Table 1). In the cryo-EM study of HTNV, approximately 10% were tubular [5], suggesting that our purification strategy provided a much greater representation of the morphological distribution of the population of virions in the supernatant.

### 3.2. Morphology of Andes Orthohantavirus

**Figure 3:**
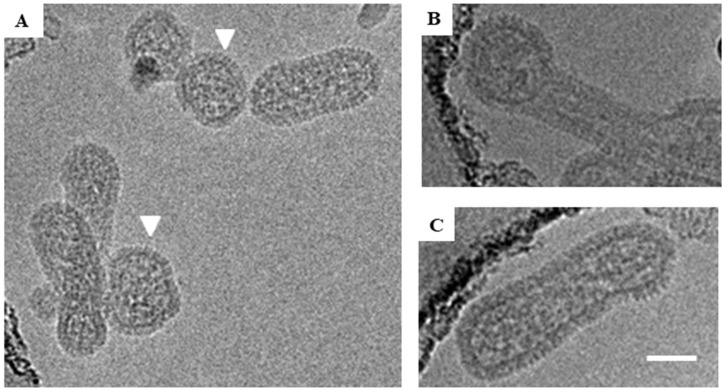
Cryo-EM images of representative ANDV particles. (**A**) Both round and tubular viral particles are shown in the image. Round particles are indicated by white triangles. The spike region of one tubular particle is marked with red box. (**B**) Irregular particle with both round and tubular portions. (**C**) Tubular particle. Scale bar = 100 nm.

**Figure 4:**
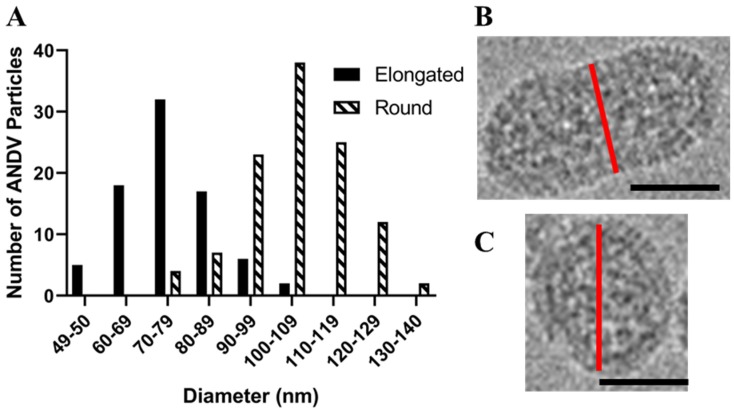
Histogram of ANDV diameters for round and tubular particles. (**A**) The diameter of 112 round and 80 tubular ANDV particles in cryo-EM images were measured and graphed in the above histogram. Panels (**B**,**C**) illustrate the axis (red line) by which diameters of elongated versus round particles were measured. Scale bars are 50 nm.

We prepared inactivated ANDV particles using our purification strategy described above and in [10] and examined them by cryo-EM. As with HNTV, we observed round (51%), tubular (37%), and irregular (12%) morphologies. The average diameter of the round virus particles was 104 nm (range, 75–140 nm), which is slightly smaller than the 119 nm mean diameter of the round HTNV particles (Figure 3A). Approximately 37% of the ANDV images had the tubular shaped particles (Figure 3C, Table 1). The most frequently observed irregular shaped morphology resembled a hybrid of the round and tubular particle (Figure 3B). Round particles had a greater diameter (105 ± 13 nm) than the cross-section of tubular particles (75 ± 11 nm) (Figure 4). The population of round and tubular particles exhibited a normal distribution (Figure 4).

### 3.3. Arrangement of Spikes on the Virus Surface and Internal RNP in ANDV

**Figure 5:**
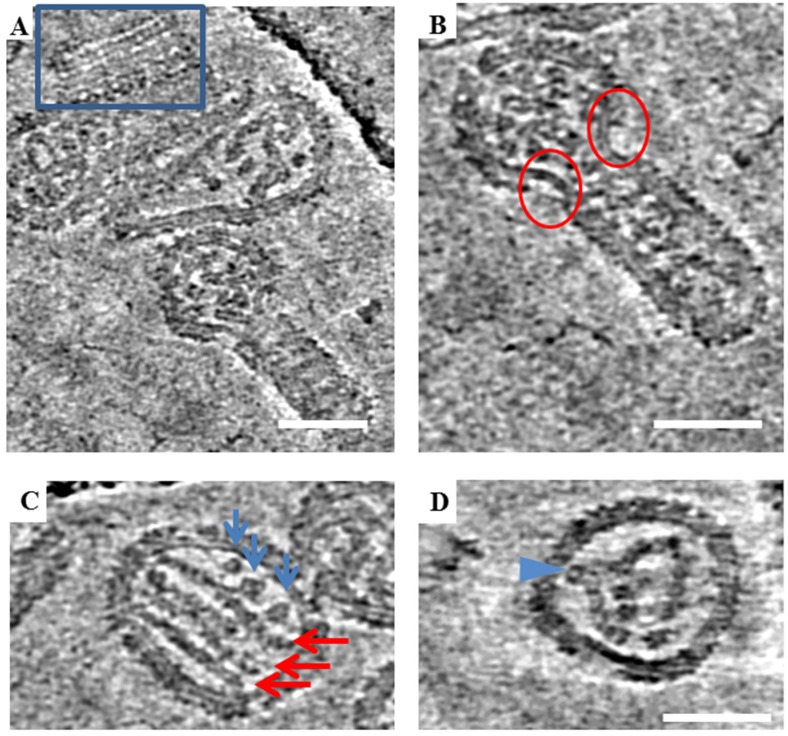
Tomographic slices showing various structural features of ANDV. (**A**) Regions boxed in blue are different slices through a tomogram showing locally ordered arrays of spikes on ANDV particles. (**B**) Regions marked by red circles show bare patches devoid of spikes on a particle with irregular morphology. Bare patches were observed to occur at the “neck” where round and tubular portions of irregular particles meet. (**C**) Representative image of parallel RNP rods in ANDV particles in a tomogram slice (red arrows) and additional densities, likely. Blue arrows point to cross sectional views through additional RNP segments. (**D**) Curved and bent RNP segment. Legend: Blue arrow indicates a point of projection and possible attachment of the RNP to the cytoplasmic domain of the transmembrane glycoprotein. Scale bars = 100 nm.

In previous cryo-EM studies, Old World hantaviruses HTNV and TULV were reported to have locally ordered arrays of glycoprotein spikes [5,6,11]. To further analyze the morphology of the New World ANDV, a very small data set consisting of two tilt series was collected and reconstructed using standard software. Tomographic slices of ANDV similarly displays a full array of glycoprotein spikes on the virion surface (Figure 5A). However, large bare patches on the virus membrane completely devoid of spikes were also observed. These patches seemed to be more numerous in particles with irregular morphology (Figure 5B). These bare patches were also noted in images of HTNV. 

Hantaviruses have internal ribonucleoprotein complexes (RNPs), formed by copies of the nucleoprotein polymerized on the three segments of the RNA genome. The RNPs have been previously observed for HTNV as 10 nm thick parallel rods [5]. Tomographic data of the ANDV virions analyzed revealed multiple parallel RNP rods inside viral particles (Figure 5C), including lengthwise 10 nm rods and additional 10 nm densities, which may correspond to RNPs observed in cross-section (Figure 5C, blue arrows). In some particles, the RNPs appear as three parallel rods whereas in others RNPs are bent and curved, projecting toward the viral membrane. These bends may represent sites where the RNP connects to the transmembrane regions of the glycoprotein spikes on the virus exterior (Figure 5D). 

### 3.4. Morphology of Two Strains of Sin Nombre Orthohantavirus 

**Figure 6:**
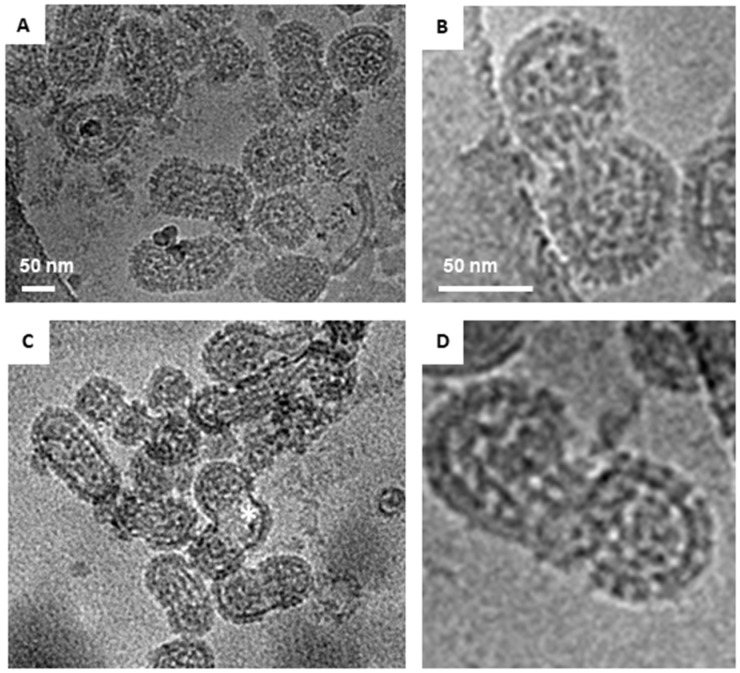
Morphology of two strains of Sin Nombre virus via cryo-EM images. (**A**) Representative image of particles of CC107 displaying round and tubular morphologies. (**B**) Zoomed in view of a particle of CC107 strain with the red boundary indicating spikes on the virus particle. (**C**) Particles of MCV strain displaying tubular and irregular (*) morphologies (**D**) Zoomed in view of a MCV particle. Scale bar = 50 nm.

Two strains of Sin Nombre virus, CC107 and MCV, were prepared using the same purification and inactivation strategy. Viruses belonging to both strains also adopted a range of round, tubular and irregular morphologies, but CC107 was more often round (Figure 6A) and MCV was more often observed to have an irregular shape (Figure 6C). Fifty-three percent of the MCV were irregular, which was the highest fraction of irregular particles amongst all the hantaviruses screened in the present study. For both strains of SNV, the average diameters were about 90 nm for round particles and 85 nm for tubular particles. The average lengths of tubular particles were 180 nm for both strains. However, the distribution of morphologies was very different in these strains (Table 1).

### 3.5. Tubular Morphology of Black Creek Canal Orthohantavirus

**Figure 7:**
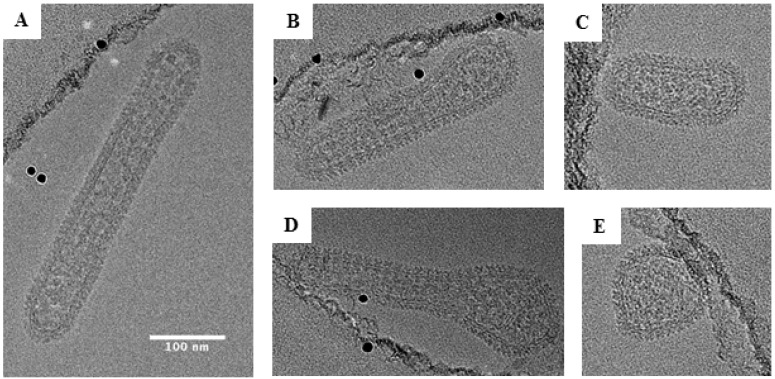
Representative cryo-EM images of Black Creek Canal virus. About 80% of the particles examined showed a tubular morphology as shown in panels (**A**–**C**). In panel (**D**), we show a representative image of an irregular virus particle. In panel (**E**), we show a representative image of a round virus particle. Scale bar: 100 nm.

The most extreme distribution of morphology was observed for BCCV in which 72% of the particles were tubular (Figure 7). Elongated BCCV particles had an average width of 79 nm (range, 50–90 nm) and an average length of approximately 239 nm (range, 140–430 nm). Some particles had lengths up to 6 times their diameter with the longest particle observed being 430 nm. This finding stands in contrast to any *Orthohantavirus* morphology reported to date. BCCV had the longest average length among all hantaviruses analyzed and is the only hantavirus in this study observed to be mostly tubular.

## 4. Discussion

New World hantaviruses have been largely unexplored structurally because of the containment required and the lack of microscopes within BSL-3 facilities. In the present study, we prepared inactivated BSL-3 viruses for cryo-EM imaging using a FEI Titan Krios microscope, outside of containment. The strategy uses two gentle, low-speed centrifugation steps of the virion preparation to concentrate the virus and remove glutaraldehyde [10]. Glutaraldehyde crosslinks the free amine groups in the protein backbone and may also react with several other functional groups [13,14]. Crosslinking is almost instantaneous at room temperature and neutral pH [15]. Glutaraldehyde undergoes self-polymerization and photooxidation if not protected from air and sunlight, [16] and hence, great care was taken to shield samples from light. We initially examined our approach using preparations of HTNV as our benchmark. A comparison of the 2D images of HTNV prepared herein as compared to virions purified by our prior sucrose-gradient method using high-speed ultracentrifugation showed that particles were similar (Figure 1) to those previously published [5]. We measured HTNV particles in cryo-EM images and noted they were in the size range described previously (120–155 nm) with spikes visible on all particles. This benchmarking suggested that the glutaraldehyde-inactivation neither disrupted virion morphology nor did the method crosslink particles. We have successfully used this method for inactivation and cryo-EM analyses of Venezuelan equine encephalitis virus [10]. Use of two successive 30% sucrose cushions for purification of the virus enabled us to obtain preparations of virus with a greater particle size distribution and morphology and confirmed the tubular structures noted in prior TEM imaging are indeed virions [17,18]. In one prior study of SNV, staining of the tubular structures for N protein was attempted but could not be confirmed [17].

This study is the first report of a cryo-EM analysis of New World hantaviruses. Our cryo-EM images and analyses suggest that there are differences in the proportions of round, elongated and irregular shapes among hantaviruses and even between strains within a single species (CC107 versus MCV). HTNV and ANDV had twice as many round particles as the North American New World hantaviruses, BCCV and SNV strains. BCCV particles had the greatest abundance of tubular morphologies of all viruses examined and the greatest lengths of any hantavirus (Figure 7A, 420 nm length). These observations are in contrast to the TEM images published by Ravkov et al. [19] in which BCCV virions budding at the apical surfaces of mammalian cells have a predominantly round morphology. Our studies are in support of findings by Goldsmith et al. where they report numerous elongated projections from the plasma membrane that they suspected were virions in the process of budding [17]. They did not confirm that these were virions. One possible reason for the observations noted in the Rakov et al. study may be that their images are based on thin sections of BCCV-infected cells while ours are focused on virions present in the supernatant. In the BCCV-infected cells, the virus particles may have been sliced along the cross section giving the initial appearance of round morphology in TEM images. Studies on respiratory syncytial virus morphology have reported that while most purified virions adopt round morphology, viruses at the budding event have predominantly tubular morphology [20,21]. Hence, in the context of the morphology we report herein, our work brings up a number of questions with respect to the entry and assembly of hantaviruses. For example, is the pleiomorphism of the viruses important for entry? How does this morphology effect the route of entry? Are round versus elongated particles more efficient in entering presumably by micropinocytosis or clathrin-mediated endocytosis? Are there certain mutations that drive the formation of elongated versus spherical particles as shown for influenza A virus? And lastly, are the elongated phenotype associated with a more virulent virus?

Two observations that we made from the tomograms of ANDV were the large size of some particles and the empty patches on the virion surface. Regarding the large size of some particles, an obvious question is whether these particles contain additional copies of the genomes. A second observation of our examination of the ANDV tomograms showed that several particles were observed to have large areas of the membrane completely devoid of spikes. This has been reported previously for HTNV [5] and TULV [6]. In some cases, these areas were up to 30–40 nm long and 10–12 tomographic sections deep (1 section = 1 pixel = 0.5 nm for 4× binned tomograms). This corresponds to an area of the membrane which could have otherwise been covered with 6–8 spikes per bare patch. This observation is similar to the estimate of 10%–30 % of the surface of TULV being bare reported by Huiskönen et al. [6]. In our study, the majority of particles with barren areas were those classified as irregular (Figure 5B). In most cases, barren surfaces were observed at the intersection of round and tubular portions of virion morphology. It is unclear if the irregularity caused the absence of surface spikes, or the absence of surface spikes caused the irregularity.

The first glimpse into New World hantavirus morphology raises many questions and new hypotheses to be tested in future studies. Since very little is known regarding how hantaviruses assemble and bud at the membrane, future research that seeks to discover how genome segments are incorporated and what determines the morphology of these pleotropic hantaviruses in the absence of a matrix protein will be critical. The 2D images showed beautiful arrays of intact spikes on all the viruses; however, these were mostly asymmetric particles not suitable for single particle averaging. Future efforts will focus on collection of larger tomography datasets for subvolume averaging of glycoprotein spikes and the internal RNPs. The RNP segments of ANDV were often observed to appear as parallel straight rods in multiple particles, with some bent and curved RNPs as well, with portions that may be in contact with membrane or cytoplasmic domains of viral glycoproteins. Cross sectional and parallel views of multiple RNPs were observed in each virion and may reflect more than one copy of the three segments in the genome. How the RNP is structured within the virion to promote assembly is an open question that may be revealed by subvolume averaging. Lastly, future research that examines the process of New World viruses budding from cells will provide important new information on the biology of these important human pathogens. 

## Figures and Tables

**Table 1:** Proportions of distinct morphologies noted in New and Old World hantaviruses.

	Species	Round (%)	Tubular (%)	Irregular (%)
Old World	HTNV (our study)	65	30	5
	HTNV (Battisti, 2011)	90	10	NR
New World	ANDV	51	37	12
	BCCV	20	72	8
	SNV (CC107)	32	48	20
	SNV (MCV)	26	21	53

Legend: NR- not reported.
